# Impact of gestational stress on postpartum endothelial function

**DOI:** 10.14814/phy2.71171

**Published:** 2026-11-10

**Authors:** Pedro Ribeiro de Souza, Juliana Arruda de Souza Monnerat, Bianca Bittencourt Luchetti, Juliana Mentzinger, Gabriel Fernandes Teixeira, Helena Naly Miguens Rocha, Fernanda Carla Ferreira de Brito, Gabriel Ferreira Lima, Nadia Alice Vieira da Motta, Anna Caldas Manhães, Vinicius Nogueira Macedo, Natalia Galito Rocha, Antonio Claudio Lucas da Nóbrega, Renata Frauches Medeiros

**Affiliations:** ^1^ Laboratory of Exercise Sciences, Department of Physiology and Pharmacology Fluminense Federal University Niteroi Brazil; ^2^ Laboratory of Nutrition and Metabolism, Department of Nutrition and Dietetics Fluminense Federal University Niteroi Brazil; ^3^ National Institute of Science and Technology–INCT Physical (In)activity and Health, CNPq Brazil; ^4^ Cardiometabology Integrative Laboratory, Department of Physiology and Pharmacology Fluminense Federal University Niteroi Brazil; ^5^ Clinical and Experimental Research Laboratory on Vascular Biology (BioVasc), Department of Physiological Sciences State University of Rio de Janeiro Rio de Janeiro Brazil; ^6^ Experimental Pharmacology Laboratory, Department of Physiology and Pharmacology Fluminense Federal University Niteroi Brazil

**Keywords:** oxidative stress, platelet aggregation, pregnancy, variable stress, vascular function

## Abstract

Variable stress (VS) is associated with increased cardiovascular risk, and its effects may be intensified during pregnancy. However, the cardiovascular impacts of VS during pregnancy on maternal health are not yet understood. Thus, the aim of this study was to evaluate the impact of VS during gestation on the endothelial function of Wistar rats in the postpartum period. Pregnant rats were divided into Control (C) and VS groups. Stress protocol was applied during the third week of gestation and lasted 8 days. After the lactation period, thoracic aorta and blood were collected for vascular reactivity assessment and platelet aggregation evaluation. Protein abundance was evaluated by western blot. Mothers who experienced VS had higher levels of corticosterone (*p* = 0.04), confirming the effectiveness of the model. VS animals showed greater sensitivity (*p* = 0.05) to phenylephrine and reduced relaxation by acetylcholine (*p* = 0.048), as well as a lower expression of eNOSp^ser1177^ (*p* = 0.007) and increased platelet aggregation (*p* = 0.02). Variable stress at the end of gestation seems to cause alterations in both the vasoconstrictor response and endothelium‐dependent vasodilation and may be related to reduced eNOS activation that persists even after lactation.

## Graphical abstract

Gestational variable stress induces persistent maternal vascular dysfunction after lactation, characterized by increased vascular sensitivity to phenylephrine, impaired endothelium‐dependent relaxation, reduced activated eNOS, and enhanced platelet aggregation.

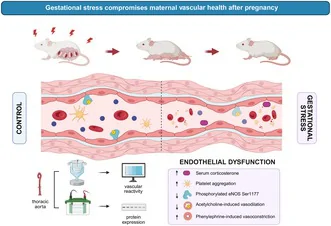

## 1 INTRODUCTION

Stress can be defined as a natural physiological response of the body to internal or external stimuli, acting as an adaptive mechanism necessary for coping with new situations. Although acute stress responses may exert protective effects in the short term, chronic exposure to one or more stressors can lead to long‐term detrimental health consequences (Rohleder, 2019).

Exposure to psychosocial stress has been associated with adverse health outcomes and an increased risk for the development of several diseases, including cardiovascular disease (CVD), insulin resistance, and cancer (Osborne et al., 2020). The positive association between stress and cardiovascular conditions has become so well established that the European Guidelines for the Prevention of Cardiovascular Diseases consider stress as an additional risk factor for individuals who already present a high risk of cardiovascular events and for those with previously diagnosed CVD (Piepoli et al., 2016).

Stress can exert deleterious effects throughout life; however, during pregnancy these repercussions may be even more pronounced. Pregnancy is characterized by profound physiological, hormonal, and cardiovascular adaptations, and the exacerbation of stress responses during this period may negatively affect both maternal and offspring health. Psychosocial, environmental, and cultural stressors during pregnancy have been shown to induce harmful effects in women and may even promote changes that persist across generations (Coussons‐Read, 2013).

In addition to neuroendocrine and autonomic changes, pregnancy represents a physiological state of high metabolic and vascular demand, in which there is a basal increase in the production of reactive oxygen species. When associated with exposure to variable stress, this scenario can favor an imbalance between pro‐oxidant and antioxidant mechanisms, culminating in exacerbated oxidative stress (Eick et al., 2018). This process is closely related to endothelial dysfunction and platelet aggregation (Ruggeri, 2002), reduced nitric oxide (NO) bioavailability, and alterations in vascular reactivity, promoting changes in tone, resistance, and blood flow (Hammadah et al., 2018).

Despite these findings, the mechanisms by which gestational stress affects maternal physiology remain incompletely understood. Stress has already been associated with increased oxidative stress biomarkers (Eick et al., 2018); however, few investigations have evaluated these effects during pregnancy and in the postpartum period.

Considering that stress is an important risk factor for the development and progression of CVD, it is believed that its impact on vascular function could be even higher during pregnancy. Therefore, the present study aimed to determine the impact of gestational variable stress on postpartum vascular function of Wistar rats.

## 2 MATERIALS AND METHODS

### 2.1 Ethics committee

This study was approved by the Ethics Committee on the Use of Animals of the Fluminense Federal University (9518170621) and is in accordance with the standards of the National Council for the control of animal experimentation.

### 2.2 Animals

Rattus novergicus, albinus variety, Wistar lineage from the LABORATORY ANIMAL CENTER of the FLUMINENSE FEDERAL UNIVERSITY (NAL/UFF) were used. During the entire experimental period, the animals were kept in polypropylene cages, in the vivarium of the UFF BIOMEDICAL INSTITUTE, in a temperature‐controlled environment (24°C ± 2°C) and adequate lighting (light and dark cycle every 12 h).

For mating, 90‐day‐old female rats were placed in collective polypropylene cages in a proportion of two females for each male. After confirmation of pregnancy using the vaginal lavage technique or the presence of a vaginal plug, pregnant females were separated into individual cages and divided into 2 groups: control and variable stress (VS). The females in the stress group performed the variable stress protocol during the third week of pregnancy (14th to the 21st gestational day), as shown in Figure 1.

**FIGURE 1:**
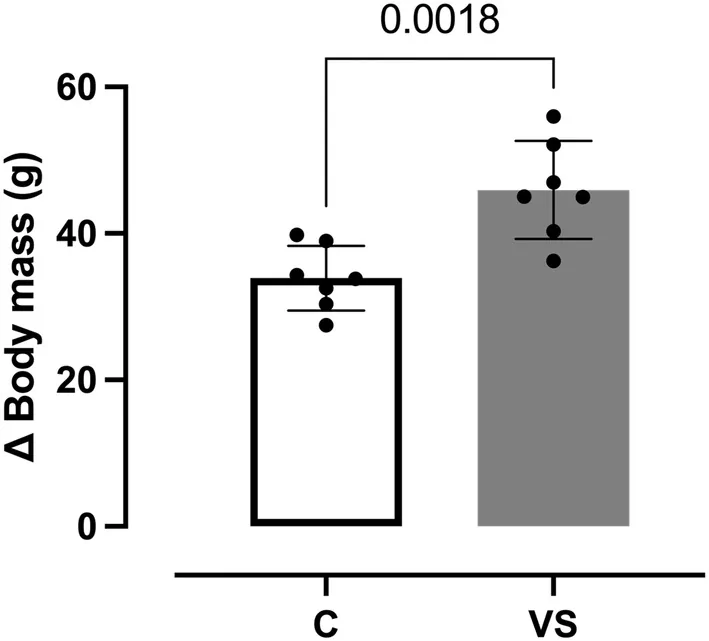
Gestational body mass gain in the control and variable stress groups. Data are presented as mean ± standard deviation. C, control group; VS, variable stress group; Δ, final body mass (g)‐ pre‐gestational body mass (g).

After the birth of the offspring, both study groups were monitored for the next 21 days, until the end of lactation, and were then euthanized by exsanguination via cardiac puncture. Pregnant rats received standard commercial food (NUVILAB® CR–formulated for rodents), provided by NAL/UFF and water ad libitum.

### 2.3 Stress protocol

Once the pregnancy was confirmed, the rats in the stress group were subjected daily to a VS protocol (Lucchetti et al., 2026; Martins et al., 2025; Mentzinger et al., 2024) during the third week of gestation, from the 14th to the 21st day of gestation. The protocol consisted of 4 different stimuli performed twice each, non‐consecutively (Table 1).

**TABLE 1:** Variable stress (VS) protocol.

Day	Stress type
1	Isolation for 60 min
2	Wet sawdust for 24 h (250 mL of tap water)
3	Cage tilt (5 cm) for 24 h
4	Deprivation of food and water for 6 h (from 3 pm to 9 pm)
5	Isolation for 60 min
6	Wet sawdust for 24 h (250 mL of tap water)
7	Cage tilt (5 cm) for 24 h
8	Deprivation of food and water for 6 h (from 3 pm to 9 pm)

### 2.4 Body mass

From T0 onwards, the weight of all rats was monitored individually, and food consumption was measured per cage using an electronic digital scale (FILIZOLA®). The change in body mass (delta) was calculated considering the final body mass (g) minus the pre‐gestational body mass (g).

### 2.5 Euthanasia and sample collection

For blood collection, the animals were fasted for 6 h, anesthetized with Ketamine (40 mg/kg) (VIRBAC LABORATORY S.A.®, São Paulo, Brazil) and Xylazine (8 mg/kg) (VIRBAC LABORATORY S.A.®, São Paulo, Brazil) and underwent metopubic incision with subsequent opening of the thoracic and abdominal cavities. Then, the blood was collected by cardiac puncture and divided into two tubes: one tube without anticoagulant to acquire serum (biochemical analysis) and one tube containing citrate to acquire plasma for platelet aggregation. To obtain plasma, the blood was centrifuged at 3.500 rpm and 4°C for 15 min (SIGMA® centrifuge), which was stored in an ultrafreezer (−80°C) for subsequent biochemical analysis. A portion of the animals from both groups had the thoracic aorta removed for vascular reactivity tests and the other portion had the aorta removed, dissected and stored in an ultrafreezer. Additionally, inguinal fat was weighed to represent subcutaneous tissue and gonadal and retroperitoneal fat to represent visceral tissue.

### 2.6 Corticosterone levels

To confirm the stress protocol effectiveness, blood samples collected on the day of the mother's euthanasia were separated for measurement of corticosterone levels by chemiluminescence (Corticosterone ELISA Kit, Item N°501320, Cayman).

### 2.7 Assessment of ex vivo platelet aggregation

Blood samples were collected by cardiac puncture into tubes containing a 3.8% trisodium citrate (9:1 v/v) solution. The platelet‐rich plasma (PRP) was prepared by centrifugation at 250 × g for 10 min at room temperature. The platelet‐poor plasma (PPP) was prepared by centrifugation of the pellet at 1500 × g for 10 min at room temperature. Platelet aggregation was monitored by the turbidimetric method, using a platelet lumi‐aggregometer (MODEL 560CA; CHRONO‐LOG CORPORATION, Havertown, PA, USA). Briefly, this method consists of platelet aggregation monitoring through transmittance. A light beam, with a wavelength equal to 240 nm, crosses the platelet suspension. The aggregometer is calibrated to the PRP corresponds to 0% transmittance and PPP corresponds to 100% transmittance. When the platelets are resting (discoid form), the platelet suspension is opaque, and the transmittance is 0%. At the time of platelet aggregation, the aggregates are formed and the light passes through the platelet suspension, quantitatively increasing the transmittance, which is proportional to the aggregation process. The platelet‐rich plasma (300 μL) was incubated at 37°C for 1 min with continuous stirring at 160 × g, and platelet aggregation was induced by ADP (0.5 μM). Platelet aggregation was expressed as the percentage of aggregation in response to ADP.

### 2.8 Vascular reactivity

The thoracic aortas were dissected and prepared for isometric tension recording. The aortas were placed in freshly prepared Krebs solution containing 119.0 mM NaCl, 14.9 mM NaHCO_3_, 11.5 mM glucose, 4.7 mM KCl, 4.2 mM KH2PO_4_, 1.2 mM MgSO_4_, and 1.6 mM CaCl_2_. The aortas were cleaned of all adherent tissue and cut into rings of approximately 3–5 mm. Each ring was suspended between two wire hooks, mounted in 10‐mL organ chambers with Krebs solution at 37°C, pH 7.4 and continuously aerated with a mixture of 95% O_2_ and 5% CO_2_ under a resting tension of 1 g. The isometric tension of the tissues was recorded by a force‐displacement transducer (INSIGHT, Brazil) connected to the software (WINDAQ DATA ACQUISITION – DATAQ INSTRUMENTS). After a stabilization period of 60–90 min, the intact endothelium aortic rings were pre‐contracted with phenylephrine (1 μM). Subsequently, the cumulative concentration–response curves to acetylcholine (ACh, 10^−9^–10^−4^ M) were obtained for the aortic rings. The relaxation curves were expressed as the percent change from the contraction induced by phenylephrine. The cumulative concentration–response curves to phenylephrine were also determined (10^−9^–10^−4^ M). Phenylephrine hydrochloride (CAS No: P6126), and acetylcholine chloride (CAS No: A6625) were purchased from Sigma Aldrich (St Louis, MO, USA). Data were expressed as maximum performance and verified by non‐linear regression using GRAPHPAD PRISM® 8.0.2 SOFTWARE (GraphPad Prism Software Inc., San Diego, CA).

### 2.9 Biochemical analyses

Capillary blood glucose was determined using a glucometer (ACCU CHEK ACTIVE®) with strips that capture the drop of hemoglobin collected through a small cut in the animal's tail. The biochemical evaluation included analysis of serum concentrations of total cholesterol (TC–K083, Bioclin), high density lipoprotein (HDL–K015, Bioclin), and triglycerides (TG–K117, Bioclin), determined with colorimetric kits. The LDL fraction of cholesterol was calculated using the Friedewald formula: [LDL] = (TC – HDL) – (TG/5). The systemic antioxidant profile was evaluated by measuring the activity of catalase (CAT–Catalase Assay Kit, n°707002, Cayman) and superoxide dismutase (SOD–Superoxide Dismutase Assay Kit, n°706002, Cayman) through radioimmunoassay.

### 2.10 Protein abundance – Western blot

The thoracic aorta was homogenized with lysis buffer containing protease and phosphatase inhibitors (Pierce™ Protease and Phosphatase Inhibitor Mini Tablets, ref.A32959, Thermo Scientific), for subsequent quantification of protein concentration using an albumin kit (Pierce™, BCA Protein Assay ref.23225, Thermo Scientific). 30 μg of protein was prepared for each tissue in sample buffer for application in a polyacrylamide gel, in which they were separated according to their molecular weight by electrophoresis and transferred from the gel to the polyvinylene fluoride membrane (PVDF – Transfer Membrane, ref.88518, Thermo Scientific), previously blocked with 3% bovine serum albumin (BSA–Bovine Serum Albumin, A7906, Sigma‐Aldrich). Then incubated overnight at 4°C with the specific primary antibodies eNOS (nitric oxide synthase) (eNOS–1:250; sc‐376751, Santa Cruz), eNOSp (phosphorylated nitric oxide synthase) (p‐eNOS^Ser1177^–1:250; ab75639, Abcam) and glutathione peroxidase (GPx–ab226004; 1:500 Abcam). Binding of primary antibodies was detected using secondary antibodies (Mouse anti‐rabbit IgG‐HRP, 1:5000, sc‐2357, Santa Cruz; Mouse IgGκ‐HRP, 1:5000, sc‐516102, Santa Cruz) and ECL (Clarity™ Western ECL Substrate; 170506; Bio‐Rad), reagents for Western Blot. The intensity of the chemiluminescent bands was quantified by densitometric analysis using ImageJ software (IMAGEJ 1.48, NATIONAL INSTITUTES OF HEALTH, USA) and the results were normalized to the expression of the endogenous control protein β‐actin (1:500; sc‐47778, Santa Cruz).

### 2.11 Statistical analysis

Results were expressed as mean ± standard deviation (SD). Data were assessed for normality using the Shapiro–Wilk test. For comparisons between groups that did not have a normal distribution, the Mann–Whitney test was used. For results with a normal distribution, the unpaired *t*‐test was used. Two‐way ANOVA was used to analyze the concentration‐response curve between groups with Tukey's post‐test. Results with *p* ≤ 0.05 were considered significant. GraphPad Prism software version 8.0 was used for these analyses.

## 3 RESULTS

### 3.1 Results of the offspring

Regarding litter size, control dams presented an average of 9.00 ± 0.71 pups per litter, whereas stressed dams presented 9.64 ± 1.86 pups per litter. No significant difference was observed between groups (*p* = 0.48). Control litters comprised 44.4% males and 40% females, while stressed litters comprised 55.6% males and 60% females. No differences in sex ratio were detected between groups (*p* = 0.85).

Concerning the birth weight of the female offspring, the group whose mother was subjected to the stress protocol had a lower birth weight (6.36 ± 0.24 g; *p* = 0.0002) when compared to the control group (6.79 ± 0.08 g). Male puppies showed the same behavior with the offspring of the mother who underwent stress, having a lower birth weight (6.61 ± 0.10 g; *p* = 0.0014) than the animals of the control mothers (7.09 ± 0.32 g).

### 3.2 Corticosterone

Mothers who underwent the stress protocol showed higher circulating corticosterone concentrations (3.38 ± 1.18 ng/mL) compared to control mothers (1.50 ± 0.35 ng/mL) (*p* = 0.04), confirming the effectiveness of stress.

### 3.3 Body mass gain

The group of mothers subjected to VS showed greater weight gain (VS = 45.94 ± 6.68 g; C = 33.89 ± 4.40 g; *p* = 0.002) from the beginning of pregnancy until the post‐lactation period (Figure 1).

### 3.4 Food consumption and caloric intake

Both groups presented similar food intake (Table 2). As expected, caloric consumption followed the same pattern.

**TABLE 2:** Food (g) and caloric (kcal) consumption during pregnancy.

	Control	Variable stress	*p* value
Food consumption (g)	461.13 ± 38.35	454.78 ± 28.65	0.73
Caloric consumption (kcal)	1549.30 ± 128.87	1528.10 ± 96.26	

*Note*: Food (g) and caloric (kcal) intake during the gestational period in rats subjected to the variable stress protocol (Stress) or not subjected to the protocol (Control). Data are presented as mean ± standard deviation.

### 3.5 Plasma glucose

Glucose values, immediately after the stress protocol, were lower in the VS group (74.57 ± 8.81 mg/dL) when compared to the C group (84.00 ± 4.28 mg/dL, *p* = 0.03) (Figure 2a). At the end of the experiment (post lactation, Figure 2b), capillary blood glucose values were similar (*p* = 0.74) between the C (102.00 ± 5.83 mg/dL) and VS (100.30 ± 12.26 mg/dL) groups.

**FIGURE 2:**
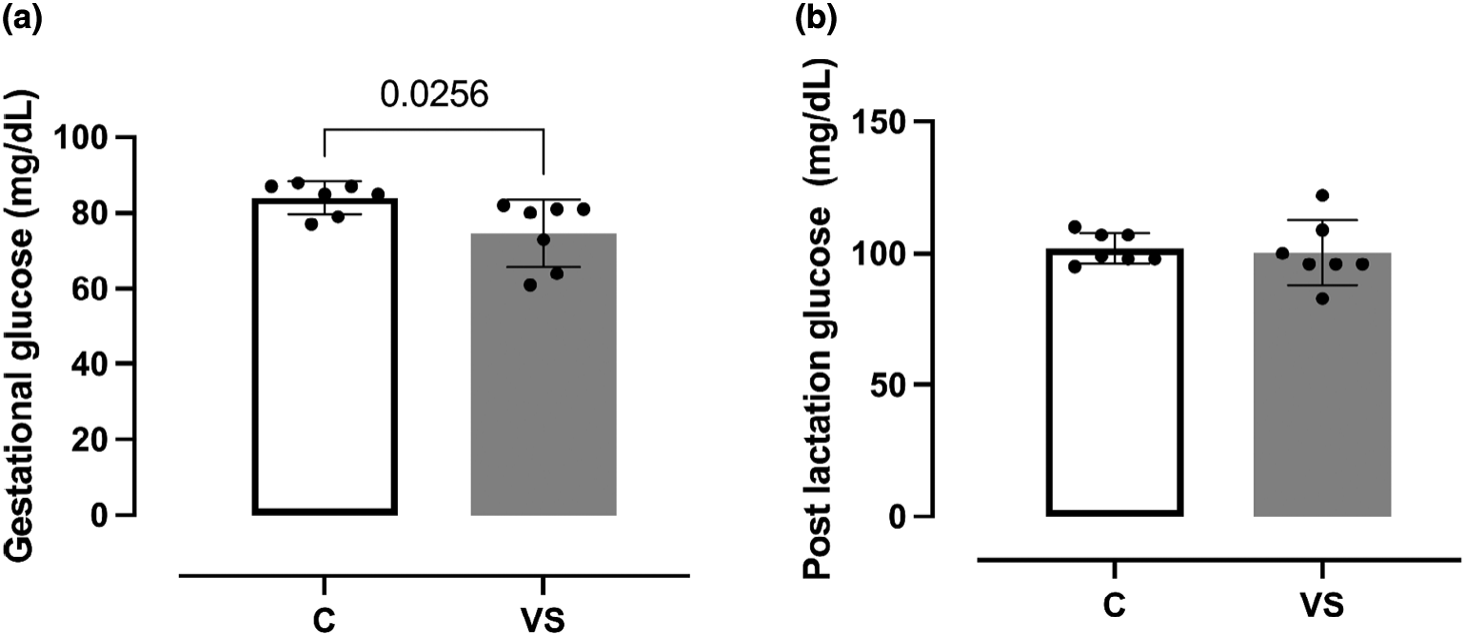
(a) Gestational glucose levels immediately after the stress protocol (b) post‐lactation. Data are presented as mean ± standard deviation. C, control group; VS, variable stress group.

### 3.6 Lipid profile

Table 3 presents the lipid profile values (total cholesterol, LDL cholesterol, HDL cholesterol, and triglycerides) of the C and VS groups. It can be observed that HDL cholesterol was lower in the VS group than the C group. There were no differences in other lipid fractions between the C and VS groups.

**TABLE 3:** Lipid profile of the experimental groups at the end of the experiment.

	Control	Variable stress	*p* value
Total cholesterol (mg/dL)	110.80 ± 29.11	103.50 ± 43.42	0.74
LDL cholesterol (mg/dL)	43.45 ± 21.46	55.63 ± 40.94	0.53
HDL cholesterol (mg/dL)	48.95 ± 7.87	35.02 ± 7.27	0.01
Triglycerides (mg/dL)	85.08 ± 54.11	65.45 ± 25.06	0.32

*Note*: Data expressed as mean ± standard deviation.

Abbreviations: HDL, high density lipoproteins; LDL, low density lipoproteins.

### 3.7 Vascular reactivity

The contractile response to phenylephrine in thoracic aortic rings is shown in Figure 3. The VS group exhibited increased sensitivity to phenylephrine (*p* = 0.05), as demonstrated by a significantly higher pEC50 value (Figure 3d). This finding is consistent with the results of the concentration–response curve (Figure 3c), indicating vascular hyperreactivity (VS Group pEC50: 7.17 ± 0.19) when compared to C group (pEC50 = 6.47 ± 0.23).

**FIGURE 3:**
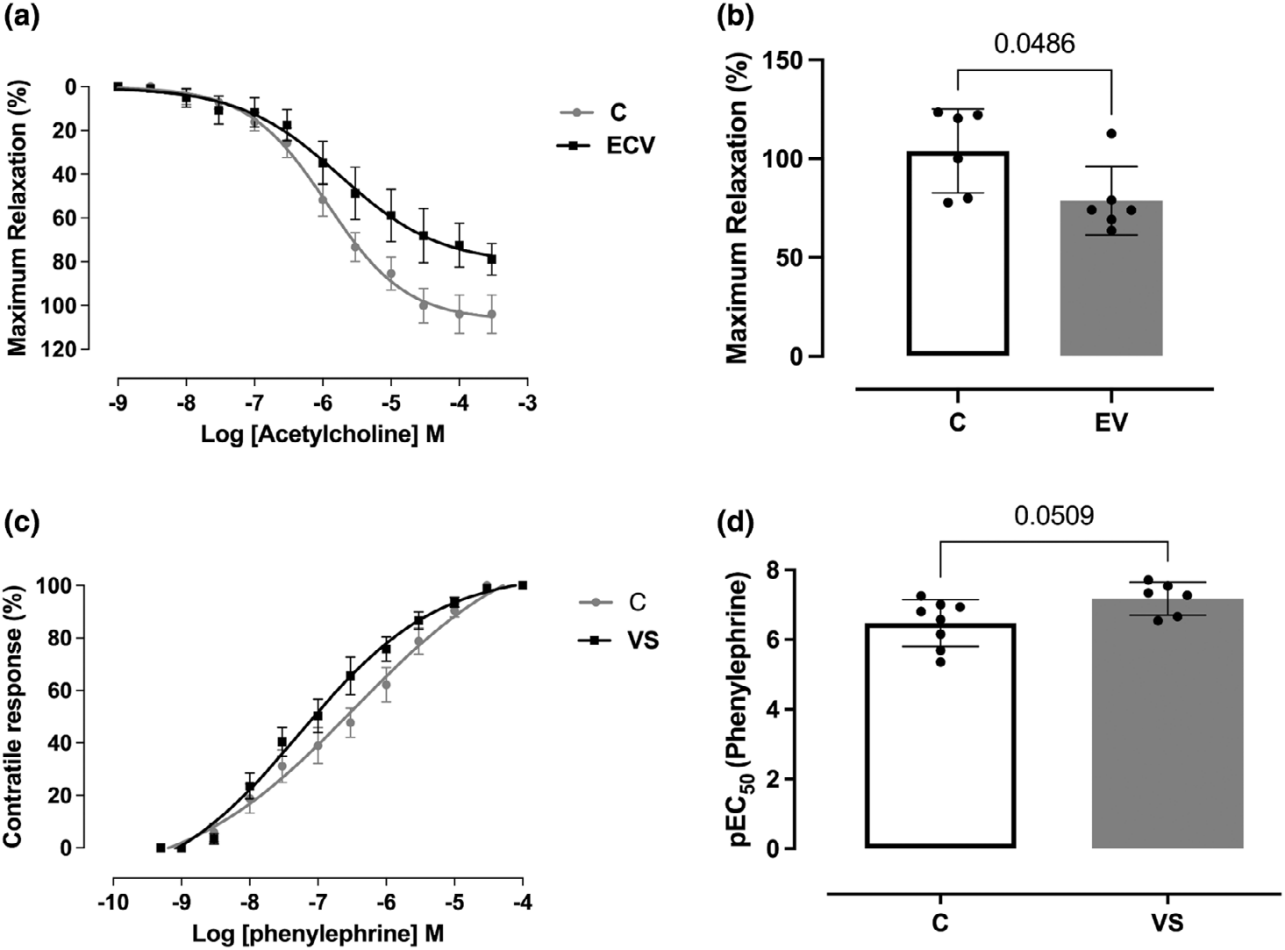
Vascular reactivity. (a) concentration–response curves to phenylephrine; (b) Maximum Acetylcholine‐Induced Relaxation (%; *p* = 0.05); (c) concentration–response curves to acetylcholine; (d) representative EC_50_ values to phenylephrine (*p* = 0.05). Values are the mean ± standard deviation from seven independent experiments. Ach, acetylcholine; C, control group; pEC50, negative logarithm of the effective concentration 50%; VS, variable stress group.

Regarding the endothelium‐dependent vascular relaxation induced by acetylcholine (Figure 3a), maximal relaxation (Figure 3b) was significantly reduced (*p* = 0.048) in mothers subjected to VS (78.79% ± 17.44%) compared with the control group (104.0% ± 21.28%), representing a 25.21% decrease in the maximal relaxation response.

### 3.8 Platelet aggregation

Figure 4 demonstrates platelet aggregation values for different concentrations of ADP (adenosine diphosphate). At 0.5 μM concentration, the VS group (47.40% ± 6.78%) showed a higher percentage of platelet aggregation (*p* = 0.02) when compared to the C group (43.00% ± 10.43%).

**FIGURE 4:**
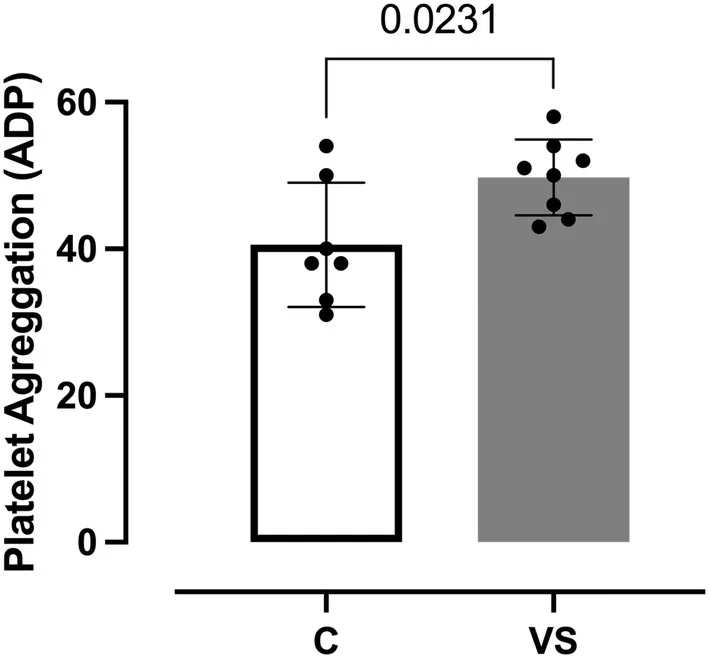
Effects of variable stress on platelet aggregation induced by ADP 0.5 μM in PRP. *p* = 0.01. Data are presented as mean ± standard deviation. ADP, adenosine diphosphate; C, control group; VS, variable stress group.

### 3.9 Plasma antioxidant stress markers

Plasma levels of oxidative stress biomarkers are presented in Figure 5. No differences were observed in SOD activity (C = 6.82 ± 0.55 U/mL, VS = 6.81 ± 0.81 U/mL; *p* = 0.9880; 6A) and CAT activity (C = 10.41 ± 5.50 nmol/min/ml; VS = 9.61 ± 3 0.88 nmol/min/ml; *p* = 0.7586; 6B) between groups.

**FIGURE 5:**
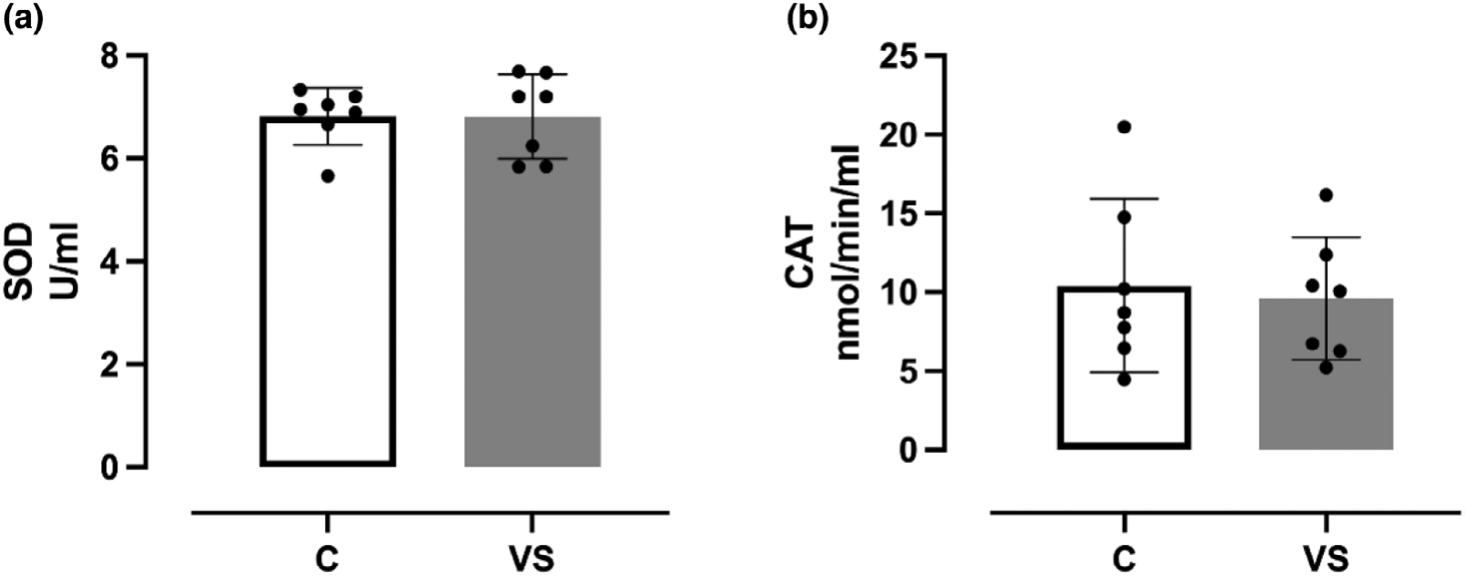
Plasma antioxidant stress marker. (a) Superoxide dismutase concentration; (b) Catalase activity. Data are presented as mean ± standard deviation. C, control group; CAT, catalase; SOD, superoxide dismutase; VS, variable stress group.

### 3.10 Expression of endothelial nitric oxide and antioxidant protein in vascular tissue

There was no difference in the expression levels of total eNOS (C: 1.00 ± 0.12; VS: 2.83 ± 2.29; *p* = 0.43–Figure 6a), but a lower expression of its active form p‐eNOS was observed; (Figure 6b) in the VS group (0.66 ± 0.33 AU) when compared to the C group (1.20 ± 0.50 AU, *p* = 0.007). No difference was observed in the expression of the antioxidant enzyme GPx (C: 1.00 ± 0.50; VS: 0.67 ± 0.33; *p* = 0.16–Figure 6c) between groups, however, lower CAT expression (C: 1.00 ± 0.87; VS: 0.17 ± 0.12; *p* = 0.02–Figure 6d) and a trend toward reduced SOD expression (C: 1.00 ± 0.76; VS: 0.25 ± 0.19; *p* = 0.06–Figure 6e) were observed in the VS group. Representative bands of protein expression in the thoracic aorta, and molecular weight and migration position of marker ladder protein in Figure 6f.

**FIGURE 6:**
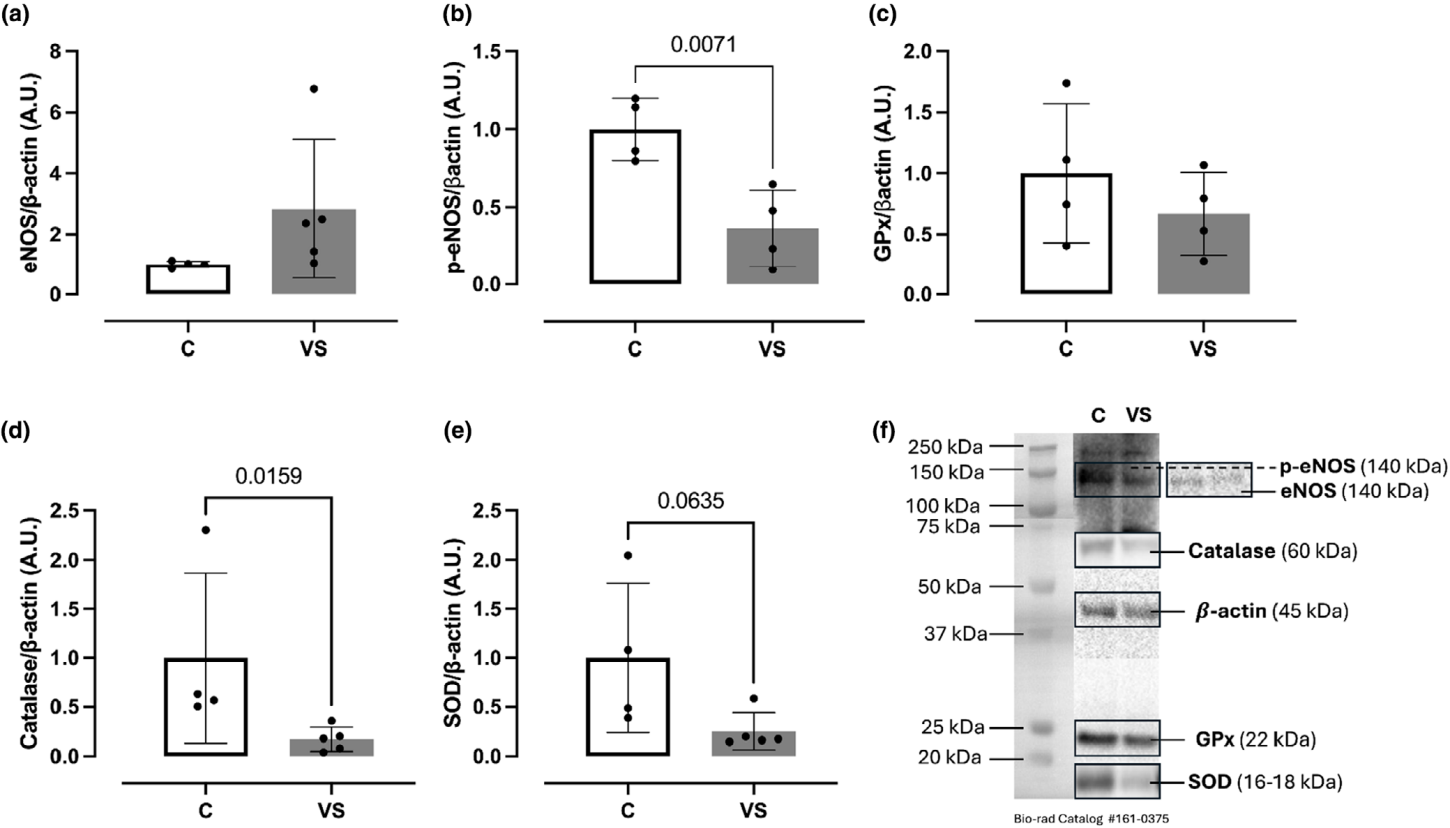
Thoracic aorta protein expression of (a) endothelial nitric oxide synthase (eNOS); (b) phosphorylated eNOS at serine 1177 (p‐eNOS^ser1177^); (c) glutathione peroxidase (GPx); (d) catalase (CAT); (e) superoxide dismutase (SOD); (f) molecular weight and migration position of marker ladder protein and representative bands of the protein expression in the thoracic aorta. Data are presented as mean ± standard deviation. C, control group; VS, variable stress group.

## 4 DISCUSSION

This study demonstrated that exposure to stress during the final period of gestation was associated with significant endocrine, metabolic, vascular, and hemostatic alterations in females. These alterations included increased corticosterone levels, greater gestational weight gain, reduced HDL cholesterol concentrations, reduced endothelial vasodilation and vasoconstrictor hyperreactivity, increased platelet aggregation, and decreased expression of the active form of eNOS and CAT. Together, these findings indicate that maternal stress affects multiple physiological systems involved in cardiovascular homeostasis.

The corticosterone levels of the animals subjected to the variable stress protocol were elevated when compared to the control group, confirming the effectiveness of the stress protocol. It is already well described in the literature that psychosocial stress can be the trigger for a series of neuroendocrine effects that culminate in changes that increase the risk of cardiovascular disease, such as increased body adiposity, mainly abdominal and visceral fat, insulin resistance and hyperinsulinemia (Lucchetti et al., 2026; Morera et al., 2019).

The greatest weight gain observed in the VS group was not accompanied by increased food intake and caloric consumption, suggesting that exposure to stress influenced the energy balance of these animals. Evidence from a previous review suggests that prolonged exposure to stressful stimuli may result in persistent disturbances in energy homeostasis, which may be detrimental and contribute to the development of metabolic disorders, including increased body weight gain (Patterson & Abizaid, 2013). Besides, psychosocial stress during pregnancy has been routinely studied for its potential association with eating behavior and/or changes in body weight; however, the results for these associations during pregnancy have been inconsistent for several reasons, including the varied stress protocols used and individual variability in gestational weight gain (Braig et al., 2020). Interestingly, exposure to stress resulted in lower blood glucose levels at the end of gestation. However, at the end of the experiment, blood glucose levels were similar between the groups. The greater production of glucocorticoids driven by the stress protocol can promote gluconeogenesis and reduce glucose uptake in adipose tissue, antagonizing the insulin response. Thus, it would be expected to increase plasma glucose and insulin in response to stress (Kuo et al., 2015). However, Quiroga et al. (2021) argue that disorders in glucose metabolism are influenced by several factors, such as the type of rodent used (rat or mouse), genetic history, type of breeding (cross or inbreeding), and the type of stress protocol used (Quiroga et al., 2021).

In contrast to glycemic levels, which became similar between the groups at the end of the experiment, HDL‐cholesterol levels were reduced in the females exposed to stress at the end of the protocol. Dyslipidemia can be caused by genetic predisposition (hereditary dyslipidemia) and other diseases, but the main modulator of lipid metabolism is lifestyle, including the consumption of a diet rich in saturated fats and simple carbohydrates, sedentary lifestyle, smoking, and stress (Francula‐Zaninovic & Nola, 2018). There are no studies available in the literature that evaluate the lipid profile of pregnant rats submitted to a VS protocol; however, in male rats, it was demonstrated that different types of stress (VS, immobilization, and restraint) are involved in the decrease in HDL‐cholesterol (Devaki et al., 2013), meaning this is probably a multi‐mechanism process involving SNS, lifestyle, and oxidative pathways, all of which are closely associated with impaired vascular reactivity (Xia & Li, 2018).

Vascular tone is maintained by the perfect balance between vasodilation and vasoconstriction, being extremely important for cardiovascular homeostasis. Impairments in the balance of this tone can increase the risk of developing CVD (Iring et al., 2019). The present results showed that the thoracic aorta of rats subjected to stress was more sensitive to α‐adrenergic vasoconstriction, an important autonomic response of the organism to specific stimuli, such as severe hypothermia and hemorrhage (Iring et al., 2019).

Some of the pathways involved in the deterioration of vascular function caused by stress have already been discussed such as an intense activation of the SNS that stimulates the production of catecholamines (norepinephrine and epinephrine) responsible for activating α adrenergic receptors, thus stimulating vasoconstriction (Kim et al., 2018). Impairments in the turnover of endothelial cells in the thoracic aorta and coronary arteries, decreased availability of NO and lower levels of nitric oxide synthase (NOS) mRNA expression (Donato et al., 2009; Osborne et al., 2020).

Additionally, for the first time in the literature, we demonstrated that mothers subjected to variable stress during gestation exhibit impaired endothelium‐dependent endothelial function, evidenced by an approximately 24% reduction in acetylcholine‐stimulated maximum vascular relaxation compared to females not exposed to stress. This finding is particularly relevant, since the literature describes that females have a greater capacity to preserve endothelial function compared to males, mainly due to the vasoprotective effects of estrogen (Brooks et al., 2018).

Estrogen contributes to the maintenance of vascular homeostasis through different mechanisms, including increased expression and activation of eNOS, resulting in greater production and bioavailability of NO. NO plays a fundamental role in the regulation of vascular tone, promoting vasodilation and protection against endothelial dysfunction (Chambliss & Shaul, 2002). In fact, previous studies demonstrate that female rodents have a greater capacity to preserve endothelial function when compared to males, even under stress conditions (Brooks et al., 2018). However, the results of the present study indicate that exposure to VS during late gestation appears to promote persistent alterations in the endothelial response.

In the present study, a decrease in p‐eNOS levels was observed in the VS group, thus impairing endothelium‐dependent vasodilation, corroborating the results of vascular reactivity presented previously. Exposure to stress is associated with impaired endothelial function, primarily due to reduced eNOS activation and/or expression, resulting in lower NO production and bioavailability (Osborne et al., 2020). Persistent activation of the hypothalamic–pituitary–adrenal axis and the sympathetic nervous system promotes increased production of reactive oxygen species (ROS), particularly through the activation of NADPH oxidase. Excess ROS rapidly reacts with NO to form peroxynitrite (ONOO^−^), reducing NO bioavailability and potentially leading to eNOS uncoupling, a condition in which the enzyme produces superoxide anion instead of NO, thereby perpetuating oxidative stress and endothelial dysfunction (Forstermann & Sessa, 2012). Consequently, there is impaired endothelium‐dependent vasodilation, increased vascular reactivity to vasoconstrictors, and a loss of vascular homeostasis, changes that contribute to the development of cardiovascular diseases (Garcia & Sessa, 2019).

The present study showed a higher percentage of platelet aggregation in the VS group. Several studies have reported that exposure to stress is associated with a prothrombotic state, characterized, among other changes, by increased platelet aggregation in rodents (Matsuhisa et al., 2014; Sandrini et al., 2017). There are no studies available in the literature that relate the effect of stress on coagulation during pregnancy; however, it is known that there is a physiological increase in thromboembolic risk in pregnancy caused by the increase in coagulation factors VII, VIII and X and fibrinogen, while fibrinolysis and protein S activity decrease, resulting in a state of blood hypercoagulation, as a physiological preparation for the hemostasis process during childbirth. The risk is even greater during the postpartum period, with pulmonary embolism being the main cause of maternal death in developed countries (Yoon, 2019). Impaired vasoconstriction, as observed in the present study, associated with a state of hypercoagulability can cause a decrease in venous return and thus facilitate the formation of thrombi (Devis & Knuttinen, 2017).

The increased vascular contractile response, reduced endothelium‐dependent relaxation, and decreased eNOS activation observed in this study may suggest the involvement of mechanisms related to oxidative stress. Exposure to the stress protocol increases the production of ROS as well as depressing antioxidant defense. Deterioration of redox homeostasis may represent an important mechanism linking psychological stress to increased cardiovascular risk (de Almeida et al., 2020).

In the present study, no significant differences were observed in SOD and CAT activity between the groups. Previous studies observed that these enzymes decrease throughout pregnancy, which may have masked stress impact, since the stressor protocol was carried out during the third trimester of pregnancy (Lekharu et al., 2014; Sharma et al., 2006; Yuksel & Yigit, 2015). However, when the expression of these antioxidant enzymes was assessed in the thoracic aorta, reduced CAT expression and a trend toward lower SOD expression were observed in the VS group. These findings support the notion that stress may induce redox imbalance, leading to increased oxidative stress and compromising vascular integrity (Handy & Loscalzo, 2022; Shaito et al., 2022).

Although the findings of the present study contribute to the understanding of the effects of gestational stress, they should be considered in light of some methodological limitations. The pharmacological inhibition of NOS was not performed, which limits the direct demonstration of the role of NO in the observed vascular effects; however, the increased phosphorylation of eNOS in Ser1177 constitutes evidence of the activation of this pathway, consistent with the vascular functional findings.

The findings of this work are highly relevant scientifically, since most studies investigating the effects of insults occurring during pregnancy focus predominantly on the offspring, while maternal repercussions remain poorly explored. In this context, our results show, originally, that exposure to stress during pregnancy promotes persistent vascular alterations, characterized by impaired endothelial function and vascular hyperreactivity. In addition to expanding knowledge about the maternal consequences of stress during pregnancy, this study provides support for future investigations of the molecular mechanisms involved in these alterations. The results also open perspectives for the development of preventive and therapeutic strategies aimed at minimizing the observed vascular damage and potentially reducing the progression to cardiovascular diseases throughout life.

## 5 CONCLUSION

This study demonstrates that exposure to variable stress at the end of gestation causes persistent alterations in vascular reactivity, observed even after the lactation period. These alterations affect both the vasoconstrictor response and endothelium‐dependent vasodilation and may be related to reduced eNOS activation and the presence of platelet aggregation in animals subjected to gestational stress.

## AUTHOR CONTRIBUTIONS

**Pedro Ribeiro de Souza:** Data curation; investigation; methodology. **Juliana Arruda de Souza Monnerat:** Conceptualization; data curation; investigation; methodology. **Bianca Bittencourt Luchetti:** Investigation; methodology. **Juliana Mentzinger:** Methodology. **Gabriel Fernandes Teixeira:** Data curation; investigation; methodology; project administration. **Helena Naly Miguens Rocha:** Data curation; investigation; methodology. **Fernanda Carla Ferreira de Brito:** Data curation; methodology; validation. **Nadia Alice Vieira da Motta:** Methodology; project administration. **Gabriel Ferreira Lima:** Methodology. **Anna Caldas Manhães:** Formal analysis. **Vinicius Nogueira Macedo:** Visualization. **Natalia Galito Rocha:** Conceptualization; data curation; formal analysis; funding acquisition; methodology; supervision. **Antonio Claudio Lucas da Nóbrega:** Conceptualization; resources; supervision. **Renata Frauches Medeiros:** Conceptualization; data curation; formal analysis; funding acquisition; investigation; methodology; project administration; resources; supervision.

## FUNDING INFORMATION

This study was supported by grants from the Carlos Chagas Filho Foundation for Research Support of the State of Rio de Janeiro (FAPERJ; E‐26/204.214/2025) and the Coordination for the Improvement of Higher Education Personnel (CAPES).

## CONFLICT OF INTEREST STATEMENT

The authors report no conflicts of interest.

## ETHICS STATEMENT

All animal procedures were approved by the Animal Ethics Committee of Fluminense Federal University under protocol number (9518170621) and were conducted in accordance with national and institutional guidelines for the care and use of laboratory animals.

## Data Availability

The data that support the findings of this study are available from the corresponding author upon reasonable request.
